# Socio-economic inequality of immunization coverage in India

**DOI:** 10.1186/2191-1991-1-11

**Published:** 2011-08-05

**Authors:** Jørgen Lauridsen, Jalandhar Pradhan

**Affiliations:** 1 Institute of Public Health - Health Economics, University of Southern Denmark, Denmark; 2 Department of Humanities and Social Sciences, National Institute of Technology, Rourkela, Orissa, India

**Keywords:** health inequality, immunization, India, decomposition, socio-economic inequality

## Abstract

To our knowledge, the present study provides a first time assessment of the contributions of socioeconomic determinants of immunization coverage in India using the recent National Family Health Survey data. Measurement of socioeconomic inequalities in health and health care, and understanding the determinants of such inequalities in terms of their contributions, are critical for health intervention strategies and for achieving equity in health care. A decomposition approach is applied to quantify the contributions from socio-demographic factors to inequality in immunization coverage. The results reveal that poor household economic status, mother's illiteracy, per capita state domestic product and proportion of illiterate at the state level is systematically related to 97% of predictable socioeconomic inequalities in full immunization coverage at the national level. These patterns of evidence suggest the need for immunization strategies targeted at different states and towards certain socioeconomic determinants as pointed out above in order to reduce socioeconomic inequalities in immunization coverage.

**JEL Classification: **I10, I12

## Background

The distributive dimension of health or health inequality has become prominent on global health policy agenda, as researchers have come to regard average health status as an inadequate summary of country's health performance [1]. Socioeconomic inequalities in child health are a major concern in developing countries to achieve the Millennium Development Goals set forth by the United Nations [2]. Yet progress towards achieving goals in reducing socioeconomic inequalities in child health may have been stymied by a critical gap in documenting and understanding trends in socioeconomic inequality in child health indicators particularly in less developed countries (endnote a). While many cross sectional studies have been performed, relatively little evidence is available regarding how socioeconomic inequalities in health have changed over time as the development process unfolded and levels of urbanization rose, women's educational attainment improved, infrastructure spread, and income and wealth increased; however, few studies have shown that socioeconomic disparities in health have in fact increased (endnote b).

In developing countries, gaps in health-related outcomes between the rich and the poor are large [3-7]. These gaps limit poor peoples' potential to contribute to the economy by reducing their capacity to function and live life to the fullest - and even to survive. The study of poor-rich inequalities in health status should not, however, solely aim to quantify their magnitude. Research should also aim to identify which population subgroups are the most disadvantaged. For this purpose, it should be possible to identify the determinants of inequalities, including those associated with age, gender, education, occupation etc. These variables have previously been identified as powerful sources of health inequalities in low and middle income countries [8,9].

A growing number of studies have examined inequalities in immunization coverage by household economic status in developing countries like India [10-14]. Many studies have assessed the level of socioeconomic inequalities in health using concentration indices and concentration curve. Though the values of concentration indices (CIs) show the degree of socio-economic inequality, it does not highlight the pathways through which inequality occurs. Decomposition of inequalities is critical to explore pathways of socioeconomic inequalities in child health.

**Figure 1:**
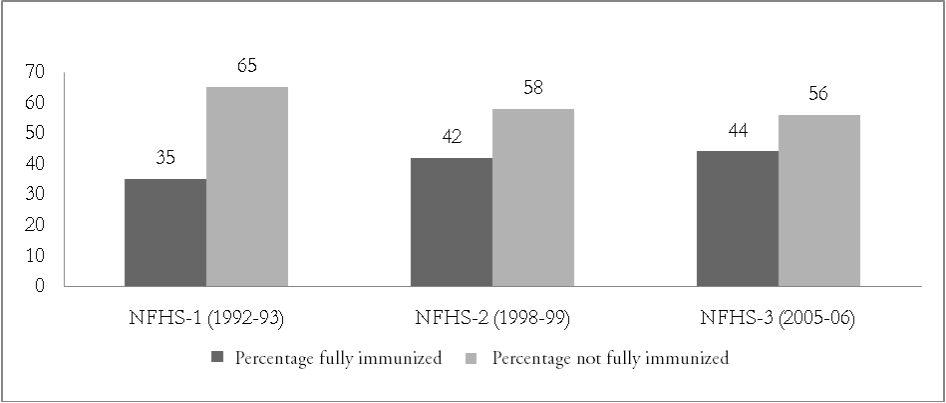
**Trend in immunization coverage, India**.

Moreover the full immunization coverage rate has only increased from 71% in 1992 to 80% in 2006 in India (Figure 1). There is a little progress from wave 2 to wave 3 of the National Family Halth Survey i.e. period from 1998-99 to 2005-06. Children not fully immunized have just declined by two percentage point i.e. from 58% to 56%. So, an intensive study is required to assess such disappointing progress in full immunization coverage.

To our knowledge, there has been virtually no study that attempted decomposition of health inequalities in the Indian context to understand such pathways. Moreover, this study also considered state level covariates along with household/individual level variables to examine the degree of contribution to the total socio-economic inequality in full immunization coverage. Given the methodological developments and the policy relevance, an attempt has been made in the present study for the first time to decompose health inequalities in terms of immunization coverage in Indian. The objective of this study is two-fold: first is to use a concentration index to quantify the socioeconomic distribution of child not fully immunized and; second is to decompose these inequalities by quantifying the contribution attributable to both household/individual covariates (i.e. economic status, education of mother, caste, residence, birth order and sex of the child) and state specific variables (i.e. poverty ratio, per-capita state domestic product, Income inequality measured in terms of Gini coefficient, % of public health spending of the total health spending, % of illiterate, % of Scheduled Tribe/Scheduled Caste population).

## Methods

Similar to previous studies initiated by Wagstaff et al. [15] we use the concentration index as our measure of relative socioeconomic inequality in immunization coverage. A concentration curve *L*(*s*) plots the cumulative proportion of the population (ranked by socioeconomic status (SES), beginning with lowest SES) against the cumulative proportion of children not being fully immunized. If *L*(*s*) coincides with the diagonal everyone is equally off. However, if *L*(*s*) lies above the diagonal, then inequality in coverage exists and favors those with high SES. The further *L*(*s*) lies from the diagonal, the greater the degree of inequality. The concentration index, *C*, is defined as twice the area between *L*(*s*) and the diagonal and takes a value of 0 when everyone is equally of regardless of SES. The minimum and maximum values of *C *are -1 and +1, respectively; these occur in the (hypothetical) situation where immunization is concentrated in the hand of the least disadvantaged and the most disadvantaged person, respectively. Thus, the larger negative value of *C*, the more the absence of full immunization concentrates among low SES groups. A computational formula for *C*, which allows for application of sample weights was given by Kakwani et al. [16] as , where  is the weighted mean of the sample, i.e. the weighted proportion not fully immunized, *N *the sample size, *y_i _* an indicator for not being fully immunized, *Wi *the sample weight of the individual (which sums to *N*) and *R_i _* the fractional rank defined according to Kakwani et al. as , i.e. the weighted cumulative proportion of the population up to the midpoint of each individual weight. Following the same authors, *C *can be conveniently computed as the weighted covariance of *y_i _* and *R_i_*, i.e. .

A straightforward way of decomposing the predicted degree of inequality into the contributions of explanatory factors was proposed by Wagstaff et al. [17]. Adapting their approach to the present case, where the health indicator is a binary variable and a logit regression specification thus applied, amounts to specifying , where  is the logit of the predicted probability of not being fully immunized and *β_k _* the logit regression coefficient for the health determinant *x_k_*. Given this linear relationship, the concentration index for  can be written as , where  is the mean of ,  the mean of *x_k _* and *C_k _* the concentration index of *x_k _* (defined analogously to *C*).

While *β_k _* measures the relationship between the health determinant *x_k _* and the logit , a more intuitive expression of the relationship between the health determinant and the probability *p_i _* is the marginal effect , where *λ*() is the logit density function. Specifically, *m_k _* expresses the average change in the probability of not being fully immunized when the health determinant *x_k _* changes one unit.

In order to assess sampling variability and to obtain standard errors for the estimated quantities, where in particular the concentration indices and the contributions, i.e. the  parts, cause troubles, we apply a "bootstrap" procedure [18,19] in a five-step manner much similar to van Doorslaer and Koolman [20]: First, sample size is inflated to allow for differences in sampling probability by dividing the sampling weights with the smallest weight and rounding to nearest integer. Second, from this expanded sample a random sub-sample of the size of the original sample is drawn with replacement. Third, the entire set of calculations as specified above are performed on this sample. Fourth, this whole process is repeated 1,000 times, each leading to replicate estimates. Fifth, using the obtained 1,000 replicates, standard deviations and *t *statistics can be computed.

## Data

Data from National Family Health Survey-3, 2005-06 [21] has been used in this study. In addition for state specific covariates data from Census 2001, Central Statistical Organisation and National Sample Survey 61st round on consumer expenditure, 2004-05 [22] are used. The National Family Health Survey-3 collected information on vaccination for all living children born in the five years preceding the survey. Information was collected from mothers for children born since 1 January, 2000 (in states that began fieldwork in 2005) and since 1 January 2001 (in states that began field work in 2006). If a card was available, the interviewer was required to carefully copy the dates on which the child received vaccinations against each disease. For vaccination not recorded on the card, the mother's report that the vaccination was or was not given was recorded. If the mother could not show a vaccination card, she was asked whether the child had received any vaccinations. If any vaccinations had been received, the mother was asked whether the child had received a vaccination against tuberculosis (BCG); diphtheria, whooping cough (pertussis), and tetanus (DPT); poliomyelitis (polio); and measles. For DPT and polio, information was obtained on the number of doses of the vaccine given to the child. Mothers were not asked the dates of vaccinations. To distinguish Polio 0 (polio vaccine given at the time of birth) from Polio 1 (polio vaccine given about six weeks after birth), mothers were also asked whether the first polio vaccine was given just after birth or later.

A binary outcome variable was calculated, namely whether or not each of the live born child aged 12-23 months received all recommended doses of vaccination or not (child fully immunized = 0; child not fully immunized = 1) (endnote c). For the core analysis we considered child not fully immunized as a dependent variable to standardize the interpretation. Two sets of independent variables (household/individual and state specific) are considered for decomposition analysis. The household/individual covariates consist of economic status (poor/non poor), education of mother (illiterate/literate), caste (scheduled caste/tribe (SC/ST)/non scheduled caste/tribe), residence (rural/urban), sex of the child (male/female), and birth order (birth order < 3, birth order 3 or more).

The state specific variables for decomposition analysis included: poverty ratio, per-capita state domestic product, income inequality measured in terms of Gini coefficient, % of public health spending of the total health spending, % of illiterate, and % of scheduled tribe/scheduled caste population.

In the National Family Health Survey-3, an index of economic status (wealth quintile) for each household was constructed using principal components analysis based on data from 109041 households. The wealth quintiles distribution was generated by applying principal components analysis on 33 household assets (endnote d). The wealth quintile distribution was used to determine poor-rich household for subsequent modelling.

For the decomposition analysis, quintiles 1 and 2, and quintiles 3, 4, and 5 were grouped together. This produced a binary variable labelled 'poor economic status', including households in the bottom 40% of economic status. Mother's education was a categorical variable with the following four levels: illiterate, primary school, guidance/high school, university. For decomposition analysis, mother's illiteracy-a binary variable- was used.

Finally, the decomposition analysis is confined to twelve possible socio-economic determinants including both household/individual and state specific variables that could explain the maximum dimension of socioeconomic inequality particularly in developing countries like India. The predictor variables of interest are i) poor economic status, ii) mother is illiterate, iii) residence in rural area, iv) sex of the child (male), v) birth order of the child (birth order 3 or more) and vi) belong to scheduled caste/scheduled tribe vii) poverty ratio, viii) per-capita state domestic product, ix) income inequality measured in terms of Gini coefficient, x) % of public health spending of the total health spending, xi) % of illiterate, and xii) % of scheduled tribe/scheduled caste population.

To take care of the non-equal probabilities of selection in different domains, a design weight was applied. The national level weight for women is calculated as, ; where W_Di _is the household design weight for the i^th ^domain is the inverse of the sampling fraction for the i^th ^domain (*f_i _= n_i_/N_i_*); R_*Hi *_is the response rate of the household interviewed; R_W*i *_is the response rate of the women interviewed. After adjustment for non response, the weights are normalized so that total number of weighted cases is equal to the total number of un-weighted cases.

## Results

Table 1 presents mean values and concentration indices of the variables selected for the study together with regression coefficients and percentage contributions to inequality in immunization of the covariates. From the column of means, it is seen that about 56 percent of the children aged 12-23 months are not fully immunized in India. Furthermore, 47 percent of the children belong to poor household economic status, and a similar proportion of children have mothers who are illiterate. A majority of the children come from rural area (74 percent).

**Table 1:** Means, concentration indices, marginal effects and contributions of covariates to inequality in immunization (N = 9582)

Variable	Mean^a^	Conc. index	Marginal effect	% Contribution
Child not fully immunized	0.564581	-0.15021***	(dep. var.)	(dep. var.)
Male child	0.532337	0.02277***	-0.13244***	0.4188***
Poor economic status	0.470733	-0.52949***	0.58956***	38.3175***
Mother is illiterate	0.477565	-0.32651***	0.85115***	34.6133***
Rural areas	0.738685	-0.17232***	0.08682***	2.8811***
Belong to Scheduled caste/tribe	0.302047	-0.24674***	0.08780***	1.7066***
Birth order 3 or more	0.407287	-0.20537***	0.35583***	7.7622***
Poverty Ratio	28.96984	-0.06000***	-0.01329***	-6.0251***
Per capita state domestic product	16433.14	0.47684***	-0.0001***	14.3303***
Income inequality (Gini Coeff)	0.329272	0.01622***	0.26994	-0.3760
% of public health spending of the total health spending	16.45547	0.04674***	0.01283***	-2.5741***
% of illiterate	36.66706	-0.05095***	0.02086***	10.1626***
% of Scheduled Caste/Scheduled Tribe population	24.43076	-0.02818***	-0.00678***	-1.2171***

Notes:

^a^: Means are weighted with population weights For concentration indices, regression coefficients and contributions, figures significantly different from zero is marked with *** (1 percent level), ** (5 percent level) and * (10 percent level).

The % contribution expresses the contribution in percentage of *Ĉ*.

The second column of Table 1 presents concentration indices for both dependent and predictive variables, which provide insights on the poor-rich distributions of immunization and the socio-economic determinants. Thus, the CI value for a child not fully immunized is -0.15021at the national level which indicates that immunization practice favors children from relatively wealthier families. Furthermore, it is seen that illiteracy of mothers, living in rural areas, belonging to scheduled cast or tribe and high birth order concentrates among the poor.

Estimated marginal effects from the regression analysis are presented in the third column of Table 1. The marginal effects indicate the association between the determinants and child health outcome indicator. The relationship between wealth and immunization coverage is evident, as children from families with poor economic status have a 59 percent higher risk of not being fully immunized. Likewise, being a child of an illiterate mother increases the risk of not being fully immunized with 85 percent, while the risks are 8 percent higher for children in rural areas, 35 percent higher for children of birth order 3 or more. Furthermore, percentage of public health spending of total health spending and percentage of illiterate population at the state level are positively related with the child health outcome indicator.

Finally, the last column of Table 1 presents the decomposition analysis of socio-economic inequalities in full immunization coverage. It is seen that the poor household economic status contributes about 38 percent of the total socioeconomic inequalities in child immunization. A major contributor is mother's illiteracy which contributes almost 34 percent to the inequality of immunization. Other important contributors are per-capita state domestic product and % of illiterate at the state level which contribute with 14 and close to 10 percent respectively. The result furthermore indicates that public health spending, income inequality and % of scheduled caste and scheduled tribe at the state level play less important role in determining the scale of health inequality in terms of child immunization.

To summarize, most predictable socioeconomic inequalities seem to arise from four socio-economic predictors: poverty itself, illiteracy of mothers, per-capita state domestic product and % of illiterate person at the state level.

## Discussion and conclusions

The study presents - to our knowledge - first time evidence on the composition of socioeconomic inequality in child health care in India in terms of children not being fully immunized. Decomposition results reveal that poor household economic status, mother's illiteracy, state domestic product and level of illiteracy at the state level contribute with about 97 percent of the total socioeconomic inequalities in full immunization coverage at the national level. Of these determinants, mother's illiteracy stands out with a contribution of about 34 percent. Furthermore, decomposition analysis of the determinants of health inequalities based on state level data, shows that neither income inequality nor the public share of health spending are significant determinants of health inequalities but per-capita state domestic product and % of illiterate population explains about 24% of the total health inequalities in full immunization coverage.

Policy implications of these results may be that health intervention strategies aiming at reducing socioeconomic inequality in immunization coverage could helpfully benefit from being supplemented with strategies aiming at reducing poverty and illiteracy in particular. Finally, intensive community level analysis is required to understand the pathways of health inequalities in full immunization coverage at the state level.

## Competing interests

The authors declare that they have no competing interests.

## Authors' contributions

JP carried out the data collection, drafted the study, wrote the background section and contributed to the results section. JL did the statistical analysis, wrote the methods section and contributed to the results section. Both authors read and approved the manuscript.

## Endnotes

a. Numerous studies have examined the effects of socioeconomic status on child health or mortality using cross-sectional data. However, few of them have extended their findings to characterize levels of inequality, using either rate ratios or, especially, more sophisticated measures of inequality. Additional complications of extracting information on trends in socioeconomic inequalities in health from cross sectional studies are that the specific measures of socioeconomic status often differ across studies, as do the number and type of other variables that are held constant [10,5,23].

b. Cleland et al. [24] found that disparities in child survival by socioeconomic status and maternal education did not narrow from the 1970s to the 1980s in a dozen of developing countries. Wagstaff's [6] reanalysis of the result from the number of studies showed that inequality in under-five mortality increased in Bolivia, from 1994 to 1998, in Vietnam from 1993 to 1998 [25], and in Uganda from 1988 to 1995 [26].

c. Fully Immunized involves received BCG, three doses of DPT and Polio, and measles vaccines.

d. The 33 household asset variables are household electrification; type of windows; drinking water source; type of toilet facility; type of flooring; material of exterior walls; type of roofing; cooking fuel; house ownership; number of household members per sleeping room; ownership of a bank or post-office account; and ownership of a mattress, a pressure cooker, a chair, a cot/bed, a table, an electric fan, a radio/transistor, a black and white television, a colour television, a sewing machine, a mobile telephone, any other telephone, a computer, a refrigerator, a watch or clock, a bicycle, a motorcycle or scooter, an animal-drawn cart, a car, a water pump, a thresher, and a tractor.
